# Comparative Genomic Analysis Reveals the Functional Traits and Safety Status of Lactic Acid Bacteria Retrieved from Artisanal Cheeses and Raw Sheep Milk

**DOI:** 10.3390/foods12030599

**Published:** 2023-02-01

**Authors:** Ilias Apostolakos, Spiros Paramithiotis, Marios Mataragas

**Affiliations:** 1 Department of Dairy Research, Institution of Technology of Agricultural Products, Hellenic Agricultural Organization “DIMITRA”, 3 Ethnikis Antistaseos St., 45221 Ioannina, Greece; 2 Department of Food Science and Human Nutrition, Agricultural University of Athens, 75 Iera Odos St., 11855 Athens, Greece

**Keywords:** dairy, fermented foods, Genotype-Phenotype, microbiology, starter cultures

## Abstract

Lactic acid bacteria (LAB) are valuable for the production of fermented dairy products. We investigated the functional traits of LAB isolated from artisanal cheeses and raw sheep milk, assessed their safety status, and explored the genetic processes underlying the fermentation of carbohydrates. *Lactiplantibacillus plantarum* had the largest and more functional genome compared to all other LAB, while most of its protein-encoding genes had unknown functions. A key finding of our analysis was the overall absence of acquired resistance genes (RGs), virulence genes (VGs), and prophages, denoting that all LAB isolates fulfill safety criteria and can be used as starter or adjunct cultures. In this regard, the identified mobile genetic elements found in LAB, rather than enabling the integration of RGs or VGs, they likely facilitate the uptake of genes involved in beneficial functions and in the adaptation of LAB in dairy matrices. Another important finding of our study was that bacteriocins and CAZymes were abundant in LAB though each species was associated with specific genes, which in turn had different activity spectrums and identified applications. Additionally, all isolates were able to metabolize glucose, lactose, maltose, and sucrose, but *Lactiplantibacillus plantarum* was strongly associated with the fermentation of rhamnose, mannose, cellobiose, and trehalose whereas *Levilactobacillus brevis* with the utilization of arabinose and xylose. Altogether these results suggest that to fully exploit the beneficial properties of LAB, a combination of strains as food additives may be necessary. Interestingly, biological processes involved in the metabolism of carbohydrates that are not of direct interest for the dairy industry may yield valuable metabolites or activate pathways associated with beneficial health effects. Our results provide useful information for the development of new probiotic artisanal cheeses and probiotic starter cultures.

## 1. Introduction

Lactic acid bacteria (LAB) are a diverse group of Gram-positive, non-sporulating, cocci or rods, which are microaerophilic or anaerobic. They are catalase-negative and produce lactic acid as the major end-product of carbohydrate fermentation [1]. LAB form two groups based on the end fermentation products, namely the homo- and hetero-fermentative groups. Hetero-fermentative LAB are further subdivided into facultatively and obligate fermentative species [2]. LAB play an important role in fermentation, producing a variety of metabolites and substances that are responsible for the sensorial properties and preservation of the final products [3]. They are of great economic importance for the production of fermented foods and for their use as starter cultures.

The use of lactic acid bacteria in the dairy industry is a well-established approach to enhance the organoleptic characteristics of fermented milk products. Cheese is one of the most popular fermented dairy products and is the most important product of the dairy industry. It is produced from milk by the action of rennet and LAB, which are added as starter cultures. The rennet breaks down the casein into peptides and amino acids, which are further metabolized by the LAB to produce energy and the main end products, lactic acid and carbon dioxide [4]. The lactic acid causes a decrease in the pH of the cheese, which is the main factor responsible for its preservation. LAB are also responsible for the production of a variety of volatile compounds and other substances that contribute to the flavor of the cheese [4,5,6,7]. Artisanal cheeses are traditional dairy products that are produced by small-scale farmers. Artisanal cheeses are usually produced by using a limited number of starter cultures and therefore are characterized by a high level of heterogeneity in their microbial composition and diversity. Artisanal cheeses constitute a complex system of microbial interactions between milk bacteria, starter cultures, and indigenous bacteria of the dairy environment [5].

With regard to their microbiological safety, LAB have the Qualified Presumption of Safety (QPS) status by the European Food Safety Authority (EFSA) and are generally recognized as safe (GRAS) in the USA [8]. In addition to technological properties, the use of LAB as food additives has increased in recent years due to their beneficial effects on human health. Some LAB strains have been used as probiotics, which are defined as live microorganisms that, when administered in adequate amounts, confer a health benefit to the host [3,9].

Whole genome sequencing (WGS) is a powerful tool for the investigation of bacterial species, as it provides a high-resolution view of the genetic diversity of bacteria [10]. The WGS technology has been applied to the study of the diversity of LAB species, including *Lactobacillus acidophilus*, *Lactiplantibacillus plantarum*, and *Lacticaseibacillus casei* [11,12]. The genomes of LAB species have been sequenced and analyzed in several studies, and the functions of the genes in these genomes have been characterized. However, the underlying genetic basis of LAB species that drives their functional traits and fermentation patterns is still not fully explored. Additionally, few studies have investigated the LAB of Greek artisanal cheeses and raw sheep milk [13,14], which limits the possibility to exploit the beneficial properties of these bacteria. In this study, we used WGS technology to conduct a comparative genomic analysis of 51 LAB strains, belonging to six different LAB species and retrieved from traditional Greek artisanal cheeses and raw sheep milk. The scope of the study was to investigate the functional traits of the LAB collection, assess their safety status, and explore the genetic processes involved in and related to the fermentation of several carbohydrates.

## 2. Materials and Methods

### 2.1. Bacterial Strains Used in the Study

The LAB used throughout this study (*n* = 51) were isolated from sheep milk, artisanal Feta, and Kefalograviera cheeses [13,14], and deposited in the culture collection of the Dairy Research Department (DRD) of Hellenic Agricultural Organization “DIMITRA” (ELGO-DIMITRA). Storage and culture conditions were performed as described by Tsigkrimani et al. [14]. The collection included the following LAB species: *Lactiplantibacillus plantarum* (*Lb. plantarum*, *n* = 16), *Levilactobacillus brevis* (*Lb. brevis*, *n* = 11), *Lactococcus lactis* subsp. *lactis* (*Lc. lactis lactis*, *n* = 9), *Pediococcus pentosaceus* (*Pd. pentosaceus*, *n* = 5), *Leuconostoc mesenteroides* (*Ln. mesenteroides*, *n* = 5), *Latilactobacillus curvatus* (*Lb. curvatus*, *n* = 3), and *Lactococcus lactis* subsp. *cremoris* (*Lc. lactis cremoris*, *n* = 2).

### 2.2. Whole Genome Sequencing, Assembly, and Quality Control

Extraction of DNA took place with the aid of the GenElute Bacterial Genomic DNA Kit (Sigma-Aldrich, St. Louis, MO, USA) according to the instructions of the manufacturer. Quality of the DNA was assessed by agarose gel electrophoresis [15] and quantification by Qubit 2.0 (ThermoFisher Scientific, Waltham, MA, USA). Library preparation was performed as follows: DNA was randomly fragmented by sonication; the ends were polished, A-tailed and ligated with Illumina’s sequencing adapters before PCR amplification using P5 and P7 primers. The AMPure XP system (Beckman Coulter, Brea, CA, USA) was employed for purification and size selection of the PCR products. The Agilent 2100 Bioanalyzer (Agilent Technologies, Santa Clara, CA, USA) was used for assessment of library size; quantification was performed by qPCR. Sequencing of the qualified libraries was performed by Novogene Genomics Service (Novogene Co., Cambridge, UK) on the Illumina Novaseq 6000 platform (Illumina, San Diego, CA, USA) (2 × 150 bp). The FastQC v.0.11 software (Babraham Bioinformatics, Cambridge, UK) that is available in the KBase platform [16,17] was used for quality assessment of the adapter-free raw reads. Polishing of the raw reads and de novo assembling into contigs were performed with the Unicycler assembler and Pilon, available on the PATRIC v3.6.8 web platform (accessed on 1 January 2022) [18,19,20]. Organization of the contigs into scaffolds was performed by the Multi-Draft based Scaffolder (MeDuSa) v1.6 [21]. Then, scaffolds were ordered and oriented on the basis of the complete reference genomes present in the NCBI database (https://www.ncbi.nlm.nih.gov/ accessed on 10 January 2022); namely *Lb. plantarum* SK151, *Lb. brevis* NPS-QW-145, *Lc. lactis* LAC460, *Pd. pentosaceus* ATCC 25745, *Ln. mesenteroides* SRCM102733, *Lb. curvatus* JCM 1096 = DSM 20019, and *Lc. cremoris* subsp. *cremoris* KW2. Quality assessment of the contigs and scaffolds, in terms of completeness (≥95%) and contamination (≤5%), was performed with the CheckM tool v1.21 [22], available on the PATRIC v3.6.8 web platform. The Skew Index Test (SkweIT) v1.0 [23] was used to assess possible mis-assemblies after scaffolding.

### 2.3. In Silico Typing and Comparative Genomic Analysis

QUAST [24] was used to assess the assembled genomes’ quality, whereas Kraken2 [25] and TYGS [26] were used to identify LAB species. The genomes were annotated using PROKKA [27]. Functional annotation and subsystem analysis of ORFs was performed with the COG database [28] via the eggnog-mapper tool [29]. The presence of clustered Regularly Interspaced Short Palindromic Repeats (CRISPRs) was assessed with the CRISPRCasFinder tool [30], while prophages integrated in LAB genomes were detected with PHASTER [31]. Abricate [32] was used to assess the existence of resistance genes (RGs), virulence genes (VGs), mobile genetic elements (MGEs), and plasmids using the Resfinder [33], VFDB [34], MobileElementFinder [35], and PlasmidFinder [36] databases, respectively. Bacteriocins were identified with BAGEL4 [34]. Presence of Carbohydrate-active enzymes (CAZymes) was assessed with the Run_dbcan V3 tool [37], whereas Traitar [38] was used to predict the phenotypic traits of LAB, in addition to those validated in a previous study performing laboratory experiments [14]. To reveal important differences in LAB pertaining to the gene-content of CAZymes, bacteriocins, and MGEs, as well as to their phenotypic traits, cluster heatmaps were computed using as input the presence/absence patterns of these features. Validation of the heatmap clusters was done with statistical analysis for overrepresentation of these features in specific LAB species. Moreover, we assessed overrepresented Gene Ontology (GO)-terms as follows. The LAB pangenome was annotated with the respective GO terms with eggnog [29]. Next, we explored enriched GO-terms with Scoary [39] and visualized the output with GoFigure! [40]. The pangenome and core-genome alignment were computed with Roary [41]. Proteins were clustered together if their amino acid sequence identity was ≥75%. The threshold for the core-genome was set at 90%. We used FastTree [42] to generate a phylogenetic tree, which was visualized with iTOL [43]. Galaxy platform [44] was used for running some of the tools. 

### 2.4. Statistical Analysis

To establish which COG categories, MGEs, CAZymes, bacteriocins, phenotypic traits, and GO terms were significantly overrepresented in each LAB species, we used the respective presence/absence data matrices as input to Scoary [39]. The significance level (alpha) was set at 0.05. The *p*-values were adjusted with Benjamini–Hochberg’s method for multiple comparisons correction. Statistical comparison in the R programming language [45] was also conducted for the basic genomic features (genome length, GC content, and number of CDS) of LAB strains with One-way ANOVA (ANalysis Of VAriance), followed by post-hoc Tukey HSD (Honestly Significant Difference). Throughout the study, LAB strains were analyzed at the species level, thus the two *Lc. lactis cremoris* strains were analyzed together with the nine *Lc. lactis lactis* strains as one species (*Lc. lactis*).

## 3. Results and Discussion

### 3.1. Assembly Statistics and Subsystem Analysis

The strain identification presented by Tsigkrimani et al. [13] was verified by the taxonomic classification with Kraken2 and TYGS. Details of species identification, isolation source, and assembly statistics for each genome are presented in Table 1. The basic characteristics of LAB genomes were evaluated statistically. The average genome length of our collection was 2.65 Mb, with *Lb. plantarum* having the largest genome (2.98 Mb, on average). Genome lengths were overall not significantly different in pairwise comparisons except for *Lb. plantarum*, which had a significantly larger genome (0.85 Mb, on average) than *Lb. brevis* (2.20 Mb on average). GC content was 44.13% on average and we found that *Lb. plantarum* and *Lc. lactis* had richer GC content than *Pd. pentosaceus* but no difference among them. Lastly, *Lc. lactis* and *Lb. curvatus* had the highest number of CDS on average, respectively, 3217 and 3087, and both had significantly more CDS than the other LAB species, especially compared to *Pd. pentosaceus* which had the smallest CDS count (1935, on average).

**Figure 1:**
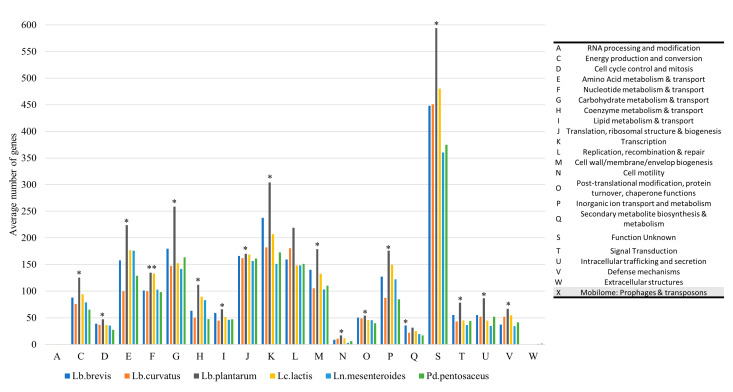
Bar chart plot showing the average number of genes per COG category for each LAB species. Abbreviation symbols for COG categories are according to the legend on the right. Species’ bars annotated with an asterisk indicate that the COG category was significantly more enriched for that species compared to all other LAB species. No genes belonging to the COG category “Mobilome: Prophages & Transposons” were identified in the isolates of our collection.

A set of CDS that collectively implement a specific biological process or structural complex is defined as a subsystem [46]. Subsystem analysis revealed the presence of 21 enriched COG categories (Figure 1). The COG category of unknown function (S) was the most enriched one, with an average of 451 CDS across LAB genomes. In this category as well as in 16/21 of COG categories overall, *Lb. plantarum* was significantly more enriched compared to all other LAB genomes (Figure 1). These findings indicate that *Lb. plantarum* has a highly functional genome and at the same time, a large part of its CDS have unknown functions that warrant further characterization [47]. Other enriched COG categories were those of transcription (K), carbohydrate metabolism and transport (G), replication and recombination (L), translation (J), and amino acid metabolism (E) having on average 209, 174, 168, 164, and 161 CDS, respectively (Figure 1). The least enriched categories were those of extracellular structures (W), present only in *Ln. mesenteroides* and *Pd. pentosaceus* (each with two CDS on average), followed by RNA processing (A), which was present only in *Lc. lactis* genomes (one CDS on average).

### 3.2. Phylogenetic Analysis and Assessment of RGs and VGs

**Figure 2:**
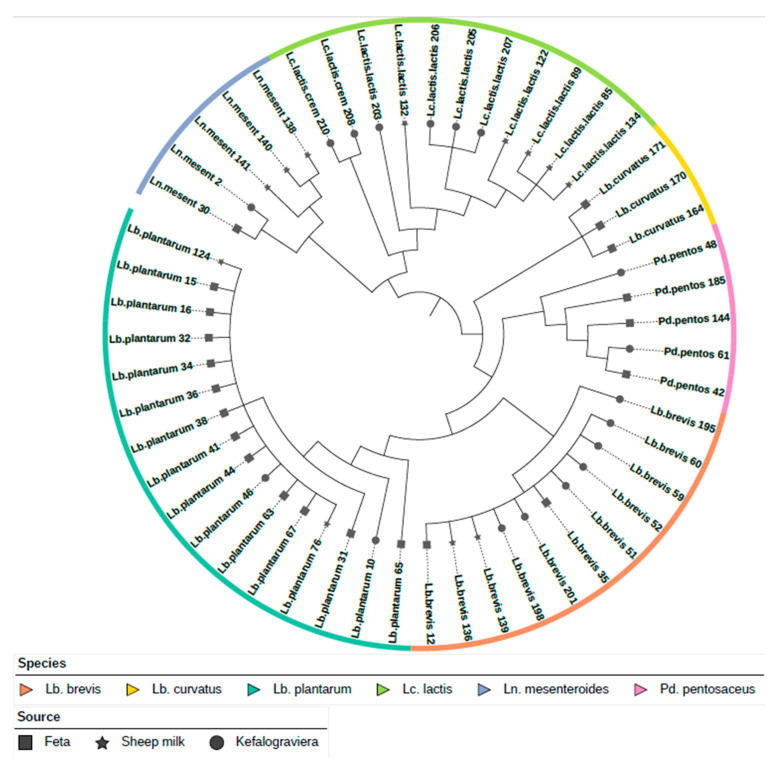
Phylogenetic tree of the 51 LAB isolates. The colored ring indicates the isolates’ species according to the legend. Symbols at the tree’s leaves denote the isolation source of each isolate according to the legend.

We found 15,585 COG clusters in the pangenome, whereas 38 COGs comprised the core-genome. The phylogenetic analysis and resulting tree indicated the genetic relatedness of LAB (Figure 2). Clusters were formed according to the species of LAB with no observable overlaps. Moreover, formation of subclusters was observed within the species clusters, which were formed according to their originating food (Feta cheese, Kefalograviera cheese, and raw sheep milk), especially for *Ln. mesenteroides* and *Lc. lactis*.

Analysis for presence of resistance and virulence genes (RGs, VGs) showed absence of VGs in all LAB, except for *Lc. lactis* genomes (*n* = 11), which contained the *lmrD* gene, an inherent, chromosomally encoded efflux pump that encodes for resistance to lincosamides in *Streptomyces lincolnensis* and *Lc. lactis*. In addition, it has been found that this gene confers resistance to bile acids in *Lc. lactis* [7,48].

### 3.3. Comparative Genomics

**Figure 3:**
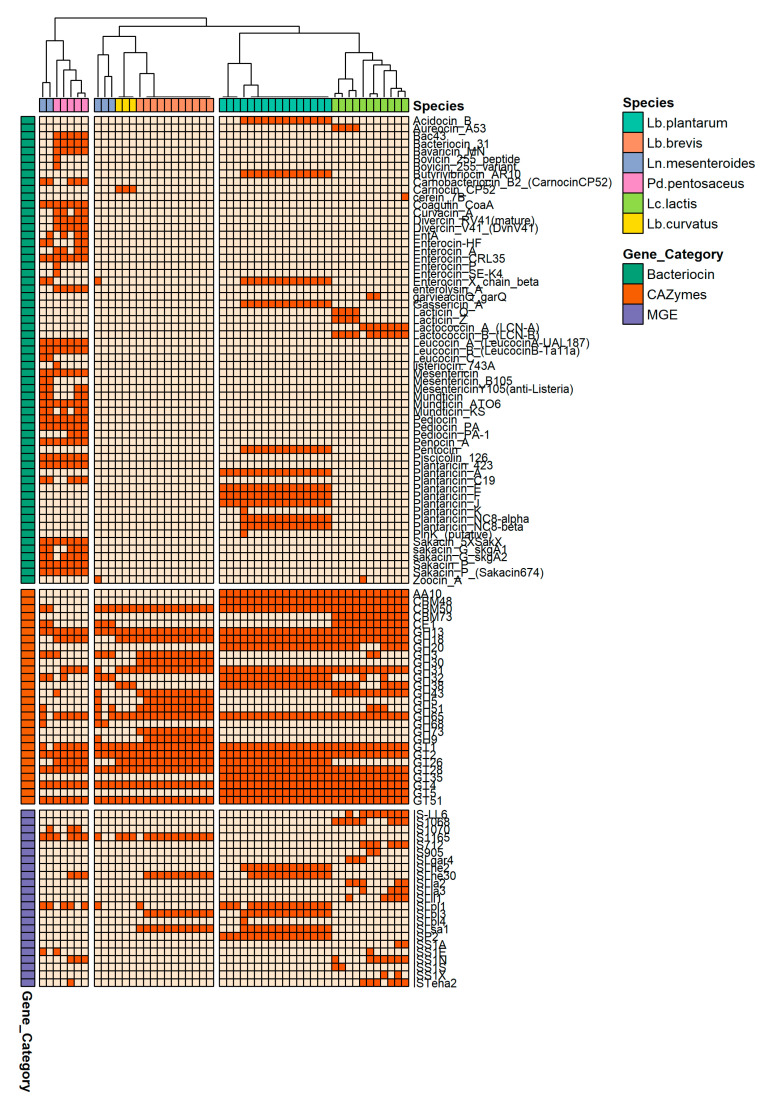
Clustered heatmap generated using a presence-absence data matrix of MGEs, CAZymes, and bacteriocins of all LAB isolates (*n* = 51). Each column represents one isolate and each row one genomic element. Isolates and genomic elements are color-coded according to the legend. Presence (dark orange) or absence (light orange) of the element in the respective isolates is shown.

A clustered heatmap was generated from the presence/absence patterns of the bacteriocins, CAZymes, and MGEs. Three major clusters were formed: *Lb. plantarum* and *Lc. lactis* formed the largest cluster, whereas *Lb. curvatus* and *Lb. brevis* clustered together with 3/5 isolates of *Ln. mesenteroides* (Figure 3). The smallest cluster contained the remaining (2/5) *Ln. mesenteroides* isolates and *Pd. pentosaceus*. This finding suggests that although phylogenetically distinct, LAB isolates share similar functional traits, most probably due to their adaptation to the specific environment of dairy products [49].

Bacteriocins are small, ribosomally synthesized peptides that are produced by bacteria as a means of inhibiting the growth of other bacteria. They are a form of antimicrobial peptide and are often specific to a particular group of bacteria or even to a single species. Bacteriocins are typically active against closely related strains but can also be active against more distantly related microorganisms [50]. They can be classified based on their mode of action, targeting the bacterial cell wall, the cytoplasmic membrane, or even the DNA/RNA of the microbes. Bacteriocins have been tested in foods as natural preservatives to inhibit pathogenic and spoilage bacteria [51].

Bacteriocins were present in all LAB species except for *Lb. brevis*. *Pediococcus pentosaceus* was the most bacteriocin-enriched species; its isolates had at least one bacteriocin-encoding gene, an average of 35 genes, and 37 unique bacteriocins (Figure 3). Overrepresentation analysis with Scoary showed strong association [Odds ratio (OR) >> 1, *p* << 0.05)] of the species with Bac43, Bacteriocin_31 (*bacA*), Bavaricin_MN, and Divercin_RV41. These bacteriocins are active against *E. faecalis*, *E. faecium*, *E. hirae*, *E. durans*, and *L. monocytogenes* [52,53]. The second most enriched species was *Ln. mesenteroides*, followed by *Lb. plantarum* with an average of 18 and 10 bacteriocins, respectively. Interestingly, we found that *Ln. mesenteroides* was not significantly associated with particular bacteriocins as most of its bacteriocin-encoding genes (*n* = 27 unique) were shared and more frequently found in *Pd. pentosaceus* (Figure 3). In contrast, *Lb. plantarum* was strongly associated (OR >> 1, *p* << 0.05) with Plantaricins (*plnA*, *plnE*, *plnF*, and *plnJ*) and Acidocin_B (*acdB*). Plantaricins have been extensively studied as natural preservatives in dairy and meat products, for their role in the inhibition of persistent bacterial infections as well their protective effect in irritable bowel syndrome disease (IBS) and urinary tract infections (UTIs) [54]. Acidocin_B (*acdB*) was first purified from *Lb. acidophilus* strain M46 and shows strong activity against *L. monocytogenes*, *Clostridium sporogenes*, and *Brochothrix thermosphacta* but is generally inactive against LAB species, hence it does not compromise the microflora stability during food fermentation [55]. Furthermore, *Lc. lactis* and *Lb. curvatus* had the least bacteriocins on average (three and one, respectively), with *Lb. curvatus* being significantly associated with carnobacteriocin B2 (*cbnB2*), a peptide active against *Listeria* and *Enterococcus* spp. [56,57].

CAZymes, short for carbohydrate-active enzymes, are a group of enzymes that catalyze the degradation, modification, or synthesis of complex carbohydrates. They are classified based on the type of carbohydrate they act on as well as the type of reaction they catalyze, such as hydrolysis, transfer, or oxidation. CAZymes play a vital role in the breakdown and utilization of carbohydrates by microorganisms. They also have potential applications in industrial processes, such as biofuel production and the production of enzymes used in food and beverage production [3]. Moreover, combining pre- and probiotics results in beneficial effects, such as the inhibition of inflammatory processes and the reduction of cholesterol levels [58]. CAZymes were ubiquitous in LAB. Overall, LAB isolates contained 17 CAZymes on average, with *Lc. lactis* being the most enriched species (*n* = 19), followed by *Lb. plantarum* and *Lb. brevis* (*n* = 18, each). In total, 28 unique CAZymes belonging to five major groups were identified. The majority of the CAZymes belonged to the Glycosyltransferase (GT) group with GT2, GT24, and GT28 being present in all LAB isolates (*n* = 51). Moreover, glycoside hydrolase (GH) 51 was present in 50/51 isolates whereas Carbohydrate-binding module (CBM) 50 in 46/51. Scoary analysis showed that *Lb. plantarum* and *Lc. lactis* were significantly associated with GT5, GT26, GT35, as well as CBM48. Of note, CBMs catalyze long CAZymes, such as glycoside hydrolases (GHs), and this process plays a key role the catabolism of complex carbohydrates such as lactose and starch [3]. In contrast, *Lb. brevis* was exclusively associated with several CAZymes (*n* = 8) of the GH group. Collectively, the aforementioned CAZymes cooperatively contribute to the dietary carbohydrate deconstruction [59].

A prophage is a bacteriophage genome that integrates in the bacterial chromosome or exists as an extrachromosomal element within the bacterial cell. Prophages that integrate in bacterial genomes often harbor resistance or virulence genes that can be transferred to the host bacterium [60]. With regard to the prophage content, none of the isolates had intact prophage regions in their genomes. The existence of CRISPR/Cas systems can help to protect bacterial genomes from prophage integration [61]. In this regard, none of the LAB isolates had robust evidence (evidence level = 4) of CRISPR sequences and *cas* genes in their genomes.

Presence of plasmids was evaluated with plasmidFinder, the detailed results are presented in Table 2. *Lactiplantibacillus plantarum* and *Lb. curvatus* had the highest plasmid content with three plasmids on average, followed by *Lc. lactis*, *Lb. brevis*, and *Ln. mesenteroides* (two plasmids on average, each). The most abundant plasmid type in these strains was a rep28 plasmid, initially reported in *Lb. plantarum* strain P-8 (NCBI accession No: CP005948) [62]. A similar distribution pattern was observed for MGEs (Figure 3). *ISLsa1* (IS30 family), the most abundant insertion sequence (IS) element, was significantly associated with *Lb. brevis* and *Lb. plantarum*. Interestingly, this IS was first reported in the meat-borne *Latilactobacillus sakei* strain 23K and is presumably involved in the translocation of genes responsible for resilience during the harsh conditions of food processing, such as fluctuating redox and oxygen levels [63]. In contrast, *Lc. lactis* was associated with *ISLL6* (IS3 family), an inherent IS element that together with *IS982* make up 1 in 10 genes encoded in on lactococcal plasmids. These IS elements have been linked with essential functional roles in dairy products, such as the production of bacteriocins, carbohydrate metabolism (CAZymes), and resistance to bacteriophage infection [64]. The predisposition of bacteria to harbor MGEs is essential in the evolution of bacterial pathogens. The presence of MGEs in the genome signifies the ability of the species to persistently harbor pathogenicity and microbial resistance factors [36,65]. Using robust alignment criteria, we showed overall absence of acquired VGs and RGs in our collection. This absence may indicate that MGEs in LAB from traditional Greek artisanal cheeses and raw sheep milk play a role in the adaptation of the bacteria in different dairy matrices [66] and are involved in beneficial functional roles such as the production of bioactive peptides and enzymes [64]. However, both aspects warrant further investigations to fully understand the function of MGEs.

### 3.4. Analysis of Phenotypic Traits

**Figure 4:**
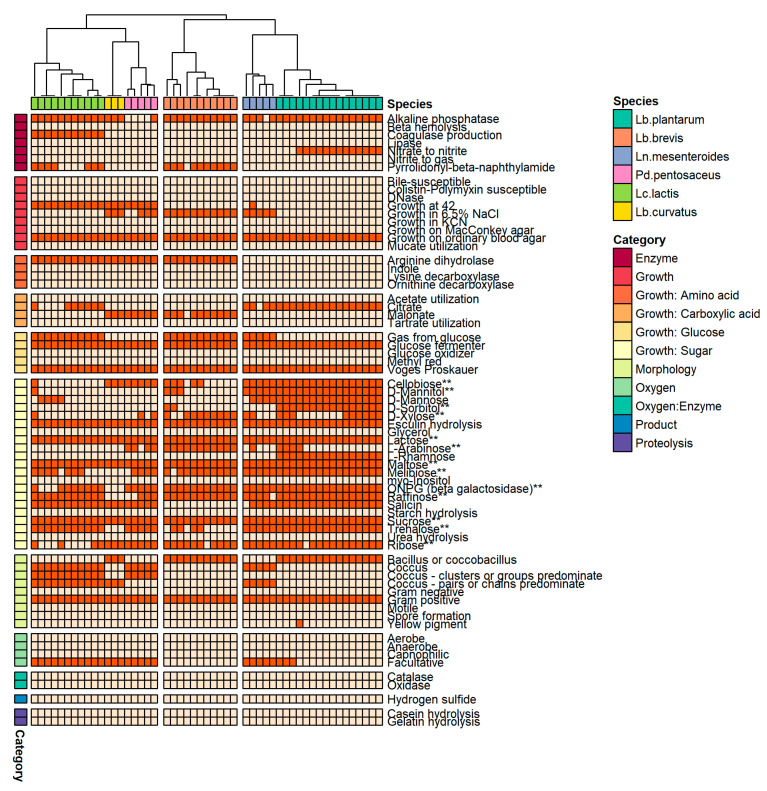
Clustered heatmap generated using a presence-absence data matrix of LAB phenotypic traits. Traits with a double asterisk (**) have been experimentally confirmed in the work of Tsigkrimani et al. (2022) [14]. Each column represents one isolate and each row one phenotypic trait. Isolates and traits are color-coded according to the legend. Presence (dark orange) or absence (light orange) of the trait in the respective isolates is shown.

The phenotypic traits evaluated experimentally in a previous work [14] were coupled with Traitar analysis for additional phenotypic characteristics and showed that, irrespective of their species, all isolates can utilize sugars such as glucose, lactose, maltose, and sucrose (Figure 4). The clustered heatmap of phenotypic traits indicated an overlap of species clusters, suggestive of shared phenotypic profiles between the studied LAB species. As with the clustered heatmap of genomic features (Figure 3), three major phenotype clusters were formed, albeit with different composition (Figure 4). The largest cluster comprised isolates of *Ln. mesenteroides* and *Lb. plantarum*. Scoary analysis indicated that *Lb. plantarum* isolates were strongly associated (OR >> 1, *p* << 0.05) with the fermentation of multiple carbohydrates (rhamnose, mannose, cellobiose, and trehalose), as well as with the sorbitol and mannitol sugar alcohols (Figure 4). In contrast, *Ln. mesenteroides* was not significantly linked to any of the assessed phenotypic traits. The second largest cluster was formed by *Lc. lactis*, *Pd. pentosaceus*, and *Lb. curvatus*. All three species were not significantly associated with specific carbohydrate fermentation. Of note, *Lc. lactis* and *Pd. pentosaceus* showed significant association with utilization of starch and malonate, suggesting that these species could be used in both dairy and vegetable fermentation [67]. The last cluster was exclusively formed by *Lb. brevis* isolates, which were significantly associated with utilization of arabinose and xylose.

In order to explore the genomic processes involved in and related to the fermentation of particular carbohydrates, we analyzed LAB genomes to identify significantly enriched Gene Ontology (GO) terms, associated with the metabolism of carbohydrates (Supplementary File S1). For cellobiose, the most overrepresented GO term clusters were (a) the *positive regulation of ribosome biogenesis* (GO:0090070) and (b) the *establishment of competence for transformation* (GO:0030420). These findings support the notion that the metabolism of cellobiose is predominantly controlled at the transcriptional level [68] while uptake of DNA from the bacterial environment (transformation) might also play a role, e.g., by the uptake of MGEs that facilitate the exchange of genes involved in carbohydrate fermentation [69]. Pathways involved in the fermentation of cellobiose might not be of direct interest for dairy products, however, it was recently reported that the same pathways drive the production of epilactose, a novel and promising prebiotic sugar which occurs from the metabolism of lactose [70]. 

Moreover, we found that the *lipid A biosynthesis* (GO:0009245) was overrepresented in LAB and able to catabolize raffinose (Supplementary File S1). Of note, it was recently reported that this pathway is upregulated in the gut microbiome of obese adults that receive whole grain wheat (WGW) products rich in oligosaccharides. In the presence of oligosaccharides such as raffinose, the lipid A biosynthesis of bacteria is upregulated. This effect, together with other modulations in the gut microbiome, lead to the amelioration of nonalcoholic fatty liver disease in obese subjects [71]. The most abundant gene in raffinose-fermenting strains involved in this pathway was *acpP* (Acyl carrier protein), encoding for a protein-carrier of the growing fatty acid chain during fatty acid biosynthesis [72].

For ribose, we found that the most overrepresented GO cluster was the *tRNA modification* (GO:0006400) comprising 20 unique GO terms (Supplementary File S1). This finding corroborates the results of McLeod et al. [73] that uptake and metabolism of ribose in *Lb. sakei* and other LAB is predominantly regulated at the transcription level. Indeed, we found that that the enzyme-encoding genes *dtd* (D-tyrosyl-tRNA(tyr) deacylase) and *truA* (tRNA pseudouridylate synthase A (pseudouridylate synthase I) were abundant in ribose-fermenting LAB strains.

Furthermore, the pathway for breakdown of arabinose to xylulose 5-phosphate and other compounds (GO:0019569) was significantly overrepresented in strains able to ferment arabinose (Supplementary File S1). The typical glycolytic routes, e.g., for glucose, in bacteria, are the Embden-Meyerhof-Parnas, the Entner-Doudoroff (ED), and the oxidative pentose phosphate pathways. However, the metabolism pathway of pentoses, such as xylose and arabinose, involve isomerases and kinases to produce xylulose 5-phosphate [74]. In this context, the gene involved in this pathway was the adolase *araD* (L-ribulose-5-phosphate 4-epimerase), a key enzyme that links the arabinose metabolic pathway to the pentose phosphate pathway and allows the bacteria to use arabinose as an energy source [75]. This gene was abundant in *Lb. brevis* strains, which were strongly associated with the fermentation of arabinose and xylose.

Lastly, the extracellular polysaccharide biosynthetic process (GO:0045226) was enriched in LAB strains, mainly belonging to *Lb. plantarum*, that were able to metabolize mannitol (Supplementary File S1). Interestingly, LAB can divert carbon resources away from glycolysis and into production of extracellular polysaccharides (EPS) [76]. EPSs have a significant role in food industrial applications; they have been employed as thickeners, stabilizers, and gelling agents in food products, like yogurt, in which they enhance the sensorial properties and “mouthfeel”. Moreover, EPSs increase the gastrointestinal transit time, thus promoting the rate of colonization of probiotic bacteria [77]. 

## 4. Conclusions

Our analysis demonstrated that *Lb. plantarum* had the largest genome and at the same time had most of the COG categories significantly more enriched compared to all other LAB genomes. Of note, most COGs of *Lb. plantarum*, and to a lesser extent of the other LAB, had unknown functions. These findings indicate that *Lb. plantarum* has a highly functional genome and at the same time a large part of its CDS is unexplored and warrant further characterization [78]. Furthermore, we showed overall absence of acquired RGs, VGs, and prophages in all LAB. According to EFSA, all of the analyzed LAB species have the QPS status as long as “the strains do not harbor any acquired antimicrobial resistance genes to clinically relevant antimicrobials” [79]. In this context, our results suggest that LAB isolated from artisanal Feta, Kefalograviera, and raw sheep milk are expected to be safe for use as starter, adjunct cultures, or as food additives in general. In this context, the identified MGEs, rather than enabling the integration of pathogenic and virulence determinants, they are likely to facilitate the uptake of genes involved in beneficial functional roles, such as the production of bioactive peptides and enzymes as well as for the adaptation of LAB in various dairy matrices [64]. Bacteriocins and CAZymes were largely ubiquitous in LAB. However, each LAB species was significantly associated with specific genes, which in turn had different activity spectrums and identified applications. Altogether these findings suggest that to fully exploit the beneficial functional properties of LAB, a combination of strains as food additives may be necessary [80]. 

The ability to ferment multiple carbohydrates is a key trait of LAB that is essential for their industrial application. All isolates were able to metabolize important dairy carbohydrates. Of note, *Lb. plantarum* and *Lb. brevis* were strongly associated with the fermentation processes of specific carbohydrates that were less abundant in the other LAB. GO analysis shed light in biological pathways involved in and related to the fermentation these carbohydrates. Interestingly, biological processes involved in the metabolism of carbohydrates that are not of direct interest for the dairy industry (e.g., cellobiose), may yield valuable metabolites or activate pathways associated with beneficial health effects.

Our results provide useful information for the further development of new probiotic artisanal cheeses and for the design of probiotic starter cultures.

## Figures and Tables

**Table 1:** Species identification and assembly statistics for all LAB genomes.

Strain ID	Microorganism	Source	Genome Size (Mb)	GC Content (%)	No. of Scaffolds	N50 (Mb)	No. of CDSs
DRD-10	*Lb. plantarum*	Kefalograviera	1.7	38.0	4.0	1.7	2445
DRD-15	*Lb. plantarum*	Feta	3.0	44.9	22.0	1.3	2435
DRD-16	*Lb. plantarum*	Feta	2.5	45.8	23.0	2.3	2449
DRD-31	*Lb. plantarum*	Feta	3.5	44.2	126.0	3.2	2170
DRD-32	*Lb. plantarum*	Feta	3.5	44.2	125.0	3.1	2450
DRD-34	*Lb. plantarum*	Feta	2.0	37.6	24.0	1.0	2449
DRD-36	*Lb. plantarum*	Feta	3.0	44.9	20.0	2.3	2453
DRD-38	*Lb. plantarum*	Feta	3.4	44.2	95.0	2.9	2449
DRD-41	*Lb. plantarum*	Feta	3.5	44.2	105.0	2.1	2450
DRD-44	*Lb. plantarum*	Feta	2.5	45.8	19.0	2.3	2454
DRD-46	*Lb. plantarum*	Kefalograviera	3.5	44.2	103.0	2.9	2450
DRD-63	*Lb. plantarum*	Feta	3.5	44.2	106.0	3.2	2143
DRD-65	*Lb. plantarum*	Feta	3.5	44.2	109.0	3.2	2147
DRD-67	*Lb. plantarum*	Feta	1.7	37.6	10.0	1.3	2147
DRD-76	*Lb. plantarum*	Sheep milk	3.5	44.2	115.0	3.2	2741
DRD-124	*Lb. plantarum*	Sheep milk	3.4	44.2	87.0	3.2	3213
DRD-208	*Lc. lactis cremoris*	Kefalograviera	1.9	37.1	14.0	1.4	3253
DRD-210	*Lc. lactis cremoris*	Kefalograviera	2.5	45.8	21.0	2.3	3254
DRD-85	*Lc. lactis lactis*	Sheep milk	2.5	45.8	22.0	2.3	2753
DRD-89	*Lc. lactis lactis*	Sheep milk	2.5	45.8	21.0	2.3	3232
DRD-122	*Lc. lactis lactis*	Sheep milk	2.5	45.8	22.0	2.3	3253
DRD-132	*Lc. lactis lactis*	Sheep milk	1.9	37.1	9.0	1.9	3264
DRD-134	*Lc. lactis lactis*	Sheep milk	3.6	44.1	227.0	3.1	3260
DRD-203	*Lc. lactis lactis*	Kefalograviera	3.0	44.9	18.0	1.0	3262
DRD-205	*Lc. lactis lactis*	Kefalograviera	3.5	44.2	115.0	3.0	3251
DRD-206	*Lc. lactis lactis*	Kefalograviera	3.5	44.2	96.0	3.2	3243
DRD-207	*Lc. lactis lactis*	Kefalograviera	2.4	34.9	19.0	1.7	3364
DRD-164	*Lb. curvatus*	Feta	2.5	34.9	26.0	2.3	2754
DRD-170	*Lb. curvatus*	Feta	2.5	34.9	30.0	0.7	3255
DRD-171	*Lb. curvatus*	Feta	3.4	44.2	92.0	3.2	3254
DRD-2	*Ln. mesenteroides*	Kefalograviera	2.6	35.0	27.0	2.2	2562
DRD-30	*Ln. mesenteroides*	Feta	2.5	34.9	31.0	1.9	2560
DRD-138	*Ln. mesenteroides*	Sheep milk	2.5	45.8	20.0	2.3	2507
DRD-140	*Ln. mesenteroides*	Sheep milk	2.2	37.5	15.0	2.1	2630
DRD-141	*Ln. mesenteroides*	Sheep milk	2.5	45.8	21.0	2.3	2458
DRD-12	*Lb. brevis*	Feta	2.0	37.7	14.0	1.1	2486
DRD-35	*Lb. brevis*	Feta	2.1	37.4	42.0	1.2	2555
DRD-51	*Lb. brevis*	Kefalograviera	2.0	37.2	17.0	1.8	2551
DRD-52	*Lb. brevis*	Kefalograviera	2.1	41.7	41.0	1.9	2506
DRD-59	*Lb. brevis*	Kefalograviera	2.1	41.7	41.0	1.9	2398
DRD-60	*Lb. brevis*	Kefalograviera	2.1	41.7	41.0	1.9	2504
DRD-136	*Lb. brevis*	Sheep milk	2.0	37.2	7.0	1.8	2253
DRD-139	*Lb. brevis*	Sheep milk	2.3	46.4	12.0	2.2	2020
DRD-195	*Lb. brevis*	Kefalograviera	2.5	45.8	20.0	2.3	2113
DRD-198	*Lb. brevis*	Kefalograviera	2.5	45.8	21.0	2.3	1710
DRD-201	*Lb. brevis*	Kefalograviera	2.6	34.9	26.0	0.5	2016
DRD-42	*Pd. pentosaceus*	Feta	2.5	34.9	25.0	1.4	2008
DRD-48	*Pd. pentosaceus*	Kefalograviera	2.5	34.9	26.0	1.3	1998
DRD-61	*Pd. pentosaceus*	Kefalograviera	2.5	34.9	15.0	2.3	1662
DRD-144	*Pd. pentosaceus*	Feta	2.5	35.4	41.0	1.8	2019
DRD-185	*Pd. pentosaceus*	Feta	2.5	35.4	43.0	1.8	1986

**Table 2:** Plasmids identified in LAB strains by the plasmidFinder tool. The number of isolates per species having a plasmid (based on the NCBI Accession column) is shown on the first column.

Species (Number of Isolates with Plasmid/Total Number of Isolates)	Plasmids	Identity (%)	Length (bp)	Note	NCBI Accession
*Lc. lactis* (5/11)	repUS4	90	1108	repA(pCI2000)	AF178424
*Lc. lactis* (8/11) *Ln. mesenteroides* (1/5)	rep32	97	1151	pli0023(pLI100)	AL592102
*Lc. lactis* (7/11)	repUS33	100	1352	repA(pGdh442)	AY849557
*Ln. mesenteroides* (5/5)	rep31	87	1132	LKI10596(LkipL4719)	CP001755
*Lb. plantarum* (11/16)	repUS73	94	1100	rep(pLBUC02)	CP002654
*Lb. plantarum* (12/16)	rep38	81	1031	rep(pLBUC03)	CP002655
*Lb. brevis* (1/11) *Pd. pentosaceus* (1/5)	rep28	92	932	repA(pCIS4)	CP003162
*Ln. mesenteroides* (1/5)	repUS72	98	1036	C27008541(pKLC4)	CP003855
*Lb. brevis* (9/11) *Lb. curvatus* (3/3) *Lb. plantarum* (12/16)	rep38	98	885	repA(LBPp1)	CP005943
*Lb. brevis* (10/11) *Lb. curvatus* (3/3) *Lb. plantarum* (13/16)	rep28	99	915	LBPp6g007(LBPp6)	CP005948
*Ln. mesenteroides* (5/5)	rep31	88	1133	LCKp400005(pLCK4)	DQ489739
*Lb. curvatus* (3/3)	repUS51	91	662	rep(pCPS49)	FN806792
*Lc. lactis* (4/11)	rep33	83	1131	rep(pSMA198)	HE613570
*Lc. lactis* (1/11)	repUS42	100	1157	repB(pVF18)	JN172910
*Lc. lactis* (1/11)	rep32	81	1168	repB(pVF22)	JN172912
*Lb. plantarum* (1/16) *Pd. pentosaceus* (1/5)	repUS64	93	956	repA(pR18)	JN601038
*Lc. lactis* (1/11)	rep33	86	1144	rep(pK214)	X92946

## Data Availability

This Whole Genome Shotgun project (BioProject number PRJNA846557, PRJNA847016, PRJNA847025, PRJNA847015, PRJNA847019, PRJNA847022, PRJNA916921) has been deposited at DDBJ/ENA/GenBank under the accession numbers JAMQJD000000000 to JAMQJS000000000 (*Lb. plantarum*), JAMRWH000000000 to JAMRWR000000000 (*Lb. brevis*), JAMRVU000000000 to JAMRVY000000000 (*Lc. lactis* subsp. *lactis* strains DRD-85, DRD-89, DRD-122, DRD-132, DRD-134), JAMRWS000000000 to JAMRWW000000000 (*Pd. pentosaceus*), JAMRWC000000000 to JAMRWG000000000 (*Ln. mesenteroides*), JAMRVZ000000000 to JAMRWB000000000 (*Lb. curvatus*), JAQGDA000000000 to JAQGDD000000000 (*Lc. lactis* subsp. *lactis* strains DRD-203, DRD-205, DRD-206, DRD-207) and JAQGDE000000000 to JAQGDF000000000 (*Lc. lactis* subsp. *cremoris* strains DRD-208, DRD-210). The version described in this paper is version JAMQJD010000000 to JAMQJS010000000 (*Lb. plantarum*), JAMRWH010000000 to JAMRWR010000000 (*Lb. brevis*), JAMRVU010000000 to JAMRVY010000000 (*Lc. lactis* subsp. *lactis*; strains DRD-85, DRD-89, DRD-122, DRD-132, DRD-134), JAMRWS010000000 to JAMRWW010000000 (*Pd. pentosaceus*), JAMRWC010000000 to JAMRWG010000000 (*Ln. mesenteroides*) and JAMRVZ010000000 to JAMRWB010000000 (*Lb. curvatus*), JAQGDA010000000 to JAQGDD010000000 (*Lc. lactis* subsp. *lactis* strains DRD-203, DRD-205, DRD-206, DRD-207) and JAQGDE010000000 to JAQGDF010000000 (*Lc. lactis* subsp. *cremoris* strains DRD-208, DRD-210).

## Supplementary Materials

The following supporting information can be downloaded at: https://www.mdpi.com/article/10.3390/foods12030599/s1, Supplementary File S1. Clusters of Gene Ontology (GO) biological processes that were overrepresented in isolates able to catabolize particular carbohydrates (cellobiose, raffinose, ribose, arabinose, and mannitol). The representative GO terms from each cluster are plotted in a two-dimensional semantic space where similar terms are placed closer to each other on the scatterplot. The size of each circle represents the number of unique GO terms found in the respective cluster.
