# Supplementary material for: Comparative Genomic Analysis Reveals the Functional Traits and Safety Status of Lactic Acid Bacteria Retrieved from Artisanal Cheeses and Raw Sheep Milk
Source: Foods. 2023 Feb 1;12(3):599. doi: 10.3390/foods12030599 (PMC9914385; doi:10.3390/foods12030599)
Supplement: Supplementary file 1 [file foods-12-00599-s001.zip › foods-2142332-supplementary File S1.pdf]

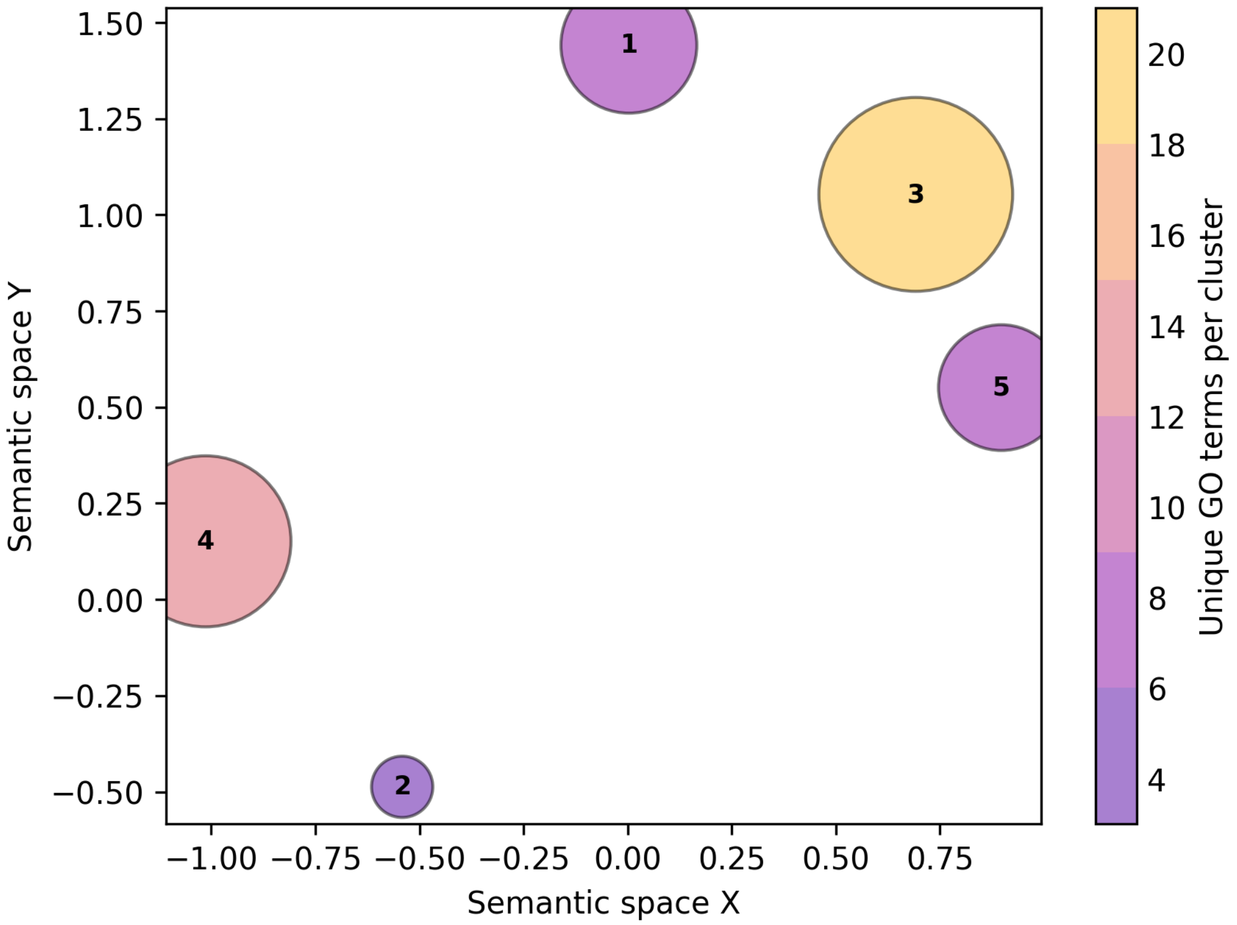

1. GO:0019359 nicotinamide nucleotide biosynthetic process
2. GO:0009294 DNA mediated transformation
3. GO:0006400 tRNA modification
4. GO:0030420 establishment of competence for transformation
5. GO:0002099 tRNA wobble guanine modification

Gene ontology (GO) biological processes significantly overrepresented in isolates able to metabolize **ribose**

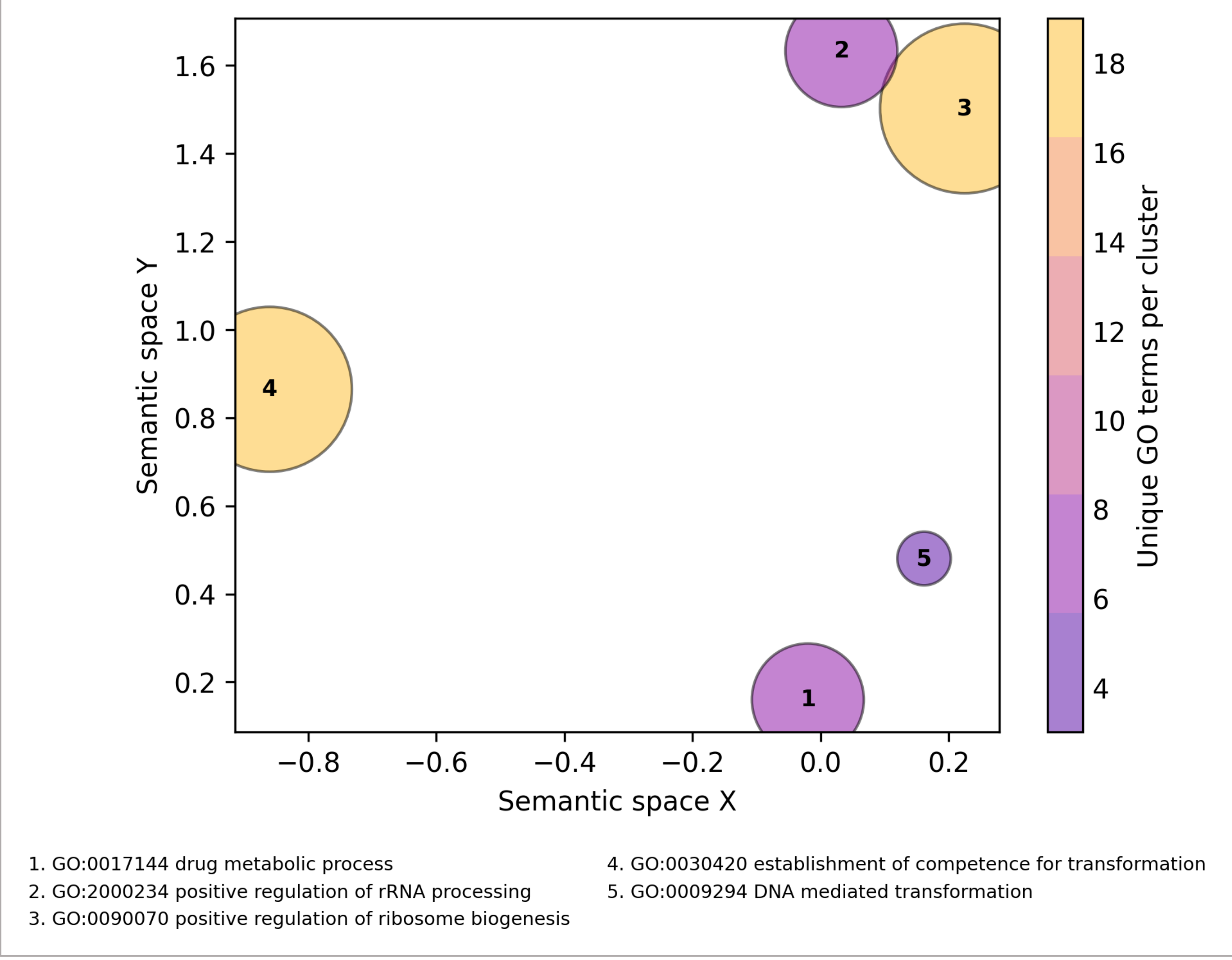

Gene ontology (GO) biological processes significantly overrepresented in isolates able to metabolize **cellobiose**

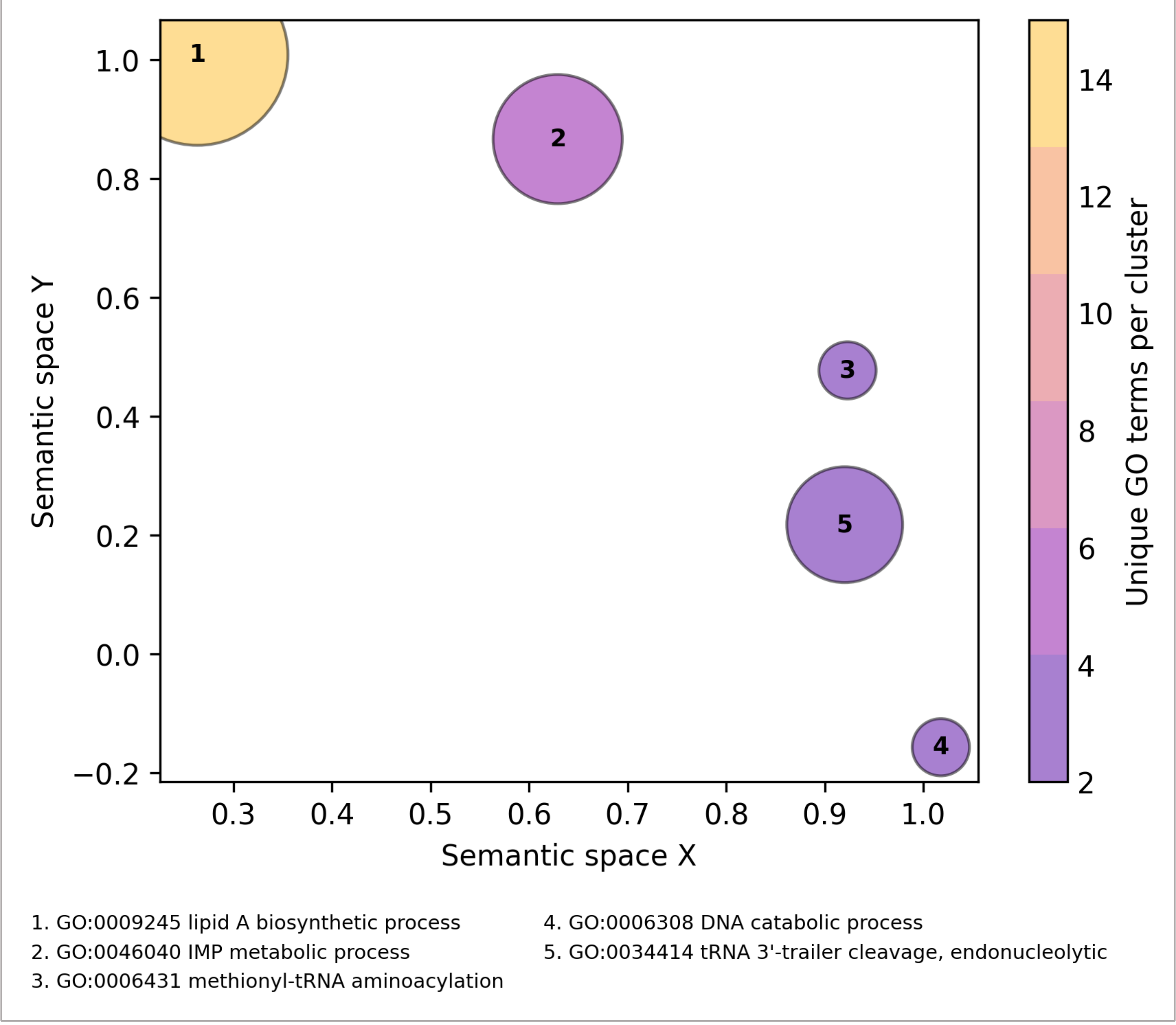

Gene ontology (GO) biological processes significantly overrepresented in isolates able to metabolize **raffinose**

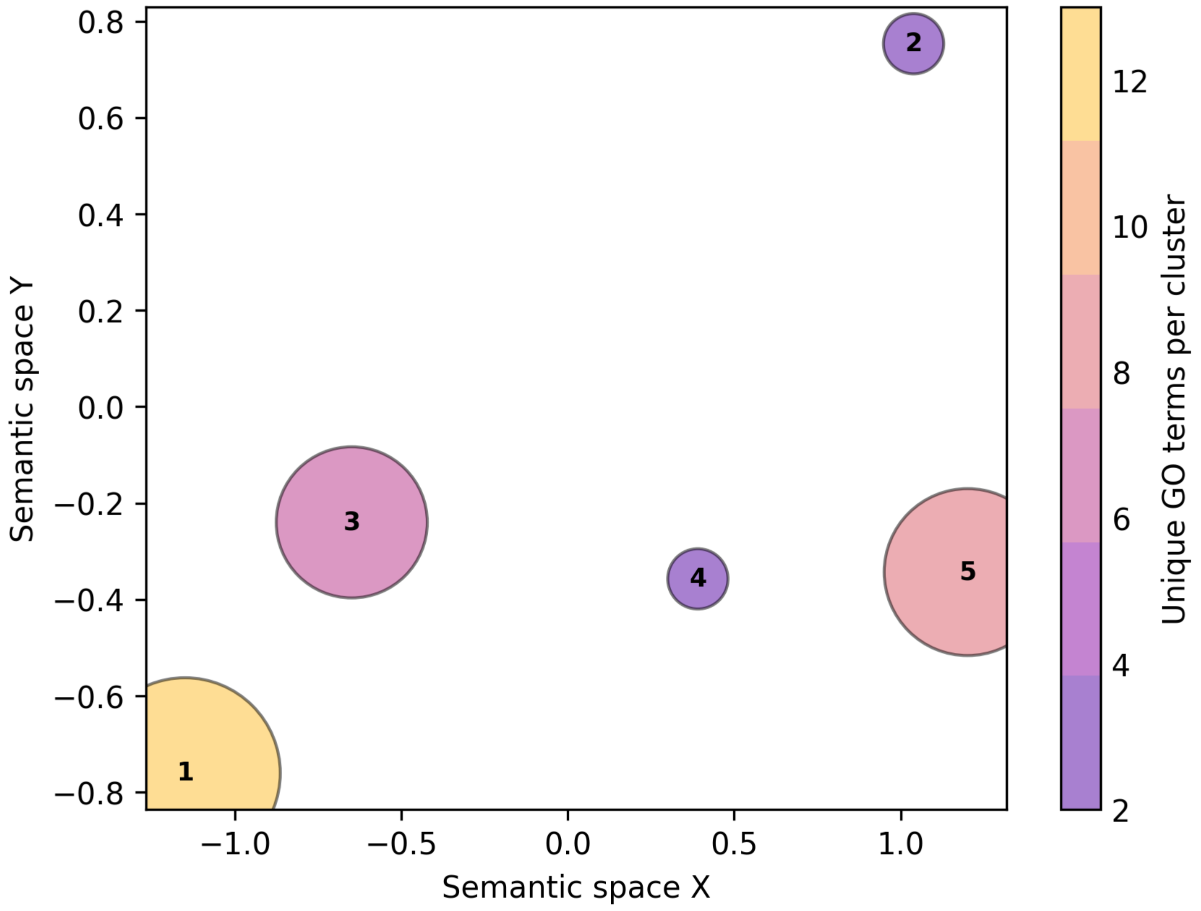

1. GO:0019569 L-arabinose catabolic process to xylulose 5-phosph...
2. GO:0046618 drug export
3. GO:0042840 D-glucuronate catabolic process
4. GO:0006413 translational initiation
5. GO:0006268 DNA unwinding involved in DNA replication

Gene ontology (GO) biological processes significantly overrepresented in isolates able to metabolize **arabinose**

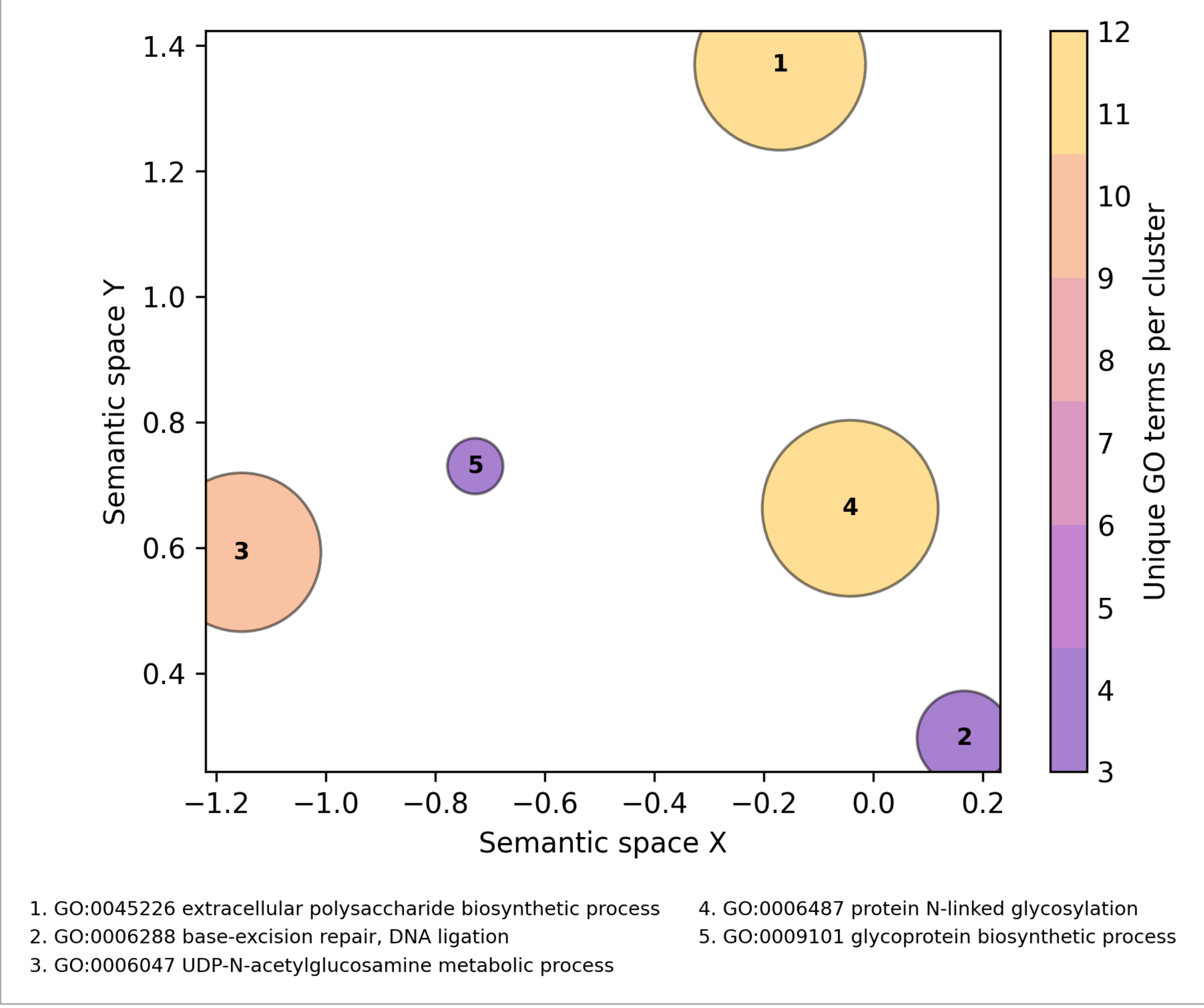

Gene ontology (GO) biological processes significantly overrepresented in isolates able to metabolize **mannitol**
